# Berkshire Medical College Commencement

**Published:** 1864-01

**Authors:** 


Berkshire Medical College Commencement.—The Berkshire Medical Institution had its annual commencement in November. The Diplomas were awarded to the graduates by the venerable President, Dr. H. H. Childs, and delivered by the Dean, Prof. Greene. We give the names, residences, and subjects of these:—

Kirk H. Bancroft, Lowell, Pneumonia; Maurice K. Bennett, Burlington, Ct., Gonorrhœa; Charles F. Couch, Pittsfield, Etiology; A. P. Folsom, Oldtown, Me, Exercise; Wm. H. Graves, New Mulford, Ct., Death; Wm. H. Gray Acton, Scorbutus; E. W. Loveland, South Hartford, N. Y., Importance of a correct Diagnosis; J. F. Niver, Cedar Hill, N. Y., Fractures; C. A. Osborne, Oneida Lake, N. Y., Puerperal Fever; Ralph Sherwood, Fairfield, Vt., Intra-Capsular Fracture Cervix Femoris; David Stephens, Addison, N. Y., Shock; R. S. Turner, Morristown, N. Y., the Human Skin; Frank Whitman, Bernardston, Coxalgia; J. J. Woodbury, North Dana, Dyspepsia; J. K. Draper, U. S. A., Quinia; B. H. Gaskill, Pancoastborough, Ohio, Physiology of Circulation.

Dr. Childs addressed the class with much feeling, complimenting them upon the high rank that they had taken as professional scholars, and invoked the Divine blessing upon their future course. The Honorary Degree of M. D. was conferred upon Drs. M. A. Patterson of Michigan, and Jonathan Brown of Massachusetts. The commencement address was given by Dr. Pliny Earle, Professor of Materia Medica, Hygiene and Psychological Medicine. The address was upon the importance of teaching the latter branch of his professorship in American Medical Schools, where it seems to have been strangely neglected—the Berkshire College having been the first, and thus far the only institution to establish a chair for instruction in this, which Prof. Earle justly styled the noblest branch of medical science—that which treats of diseases of the mind.

